# Low-density lipoprotein particle size in hepatic steatosis and metabolic syndrome

**DOI:** 10.1186/1758-5996-2-18

**Published:** 2010-03-22

**Authors:** Dal-Sik Kim, Young-Kon Kim, Do-Sung Kim, Han-Jung Chae, Tae-Sun Park, Young I Cho, Seul-Ki Jeong

**Affiliations:** 1 Department of Laboratory Medicine, Research Institute of Clinical Medicine, Chonbuk National University Medical School & Hospital, Jeonju, Jeonbuk, South Korea; 2 Department of Diagnostic Radiology, Research Institute of Clinical Medicine, Chonbuk National University Medical School & Hospital, Jeonju, Jeonbuk, South Korea; 3 Department of Pharmacology and Institute of Cardiovascular Research, Chonbuk National University Medical School, Jeonju, Jeonbuk, South Korea; 4 Department of Internal Medicine, Research Institute of Clinical Medicine, Chonbuk National University Medical School & Hospital, Jeonju, Jeonbuk, South Korea; 5 Department of Mechanical Engineering and Mechanics, Drexel University, Philadelphia, PA, USA; 6 Department of Neurology, Research Institute of Clinical Medicine, Chonbuk National University Medical School & Hospital, Jeonju, Jeonbuk, South Korea

## Abstract

**Background:**

Hepatic steatosis (HS), the most frequent liver disorder, was reported to be an independent predictor of cardiovascular disease. HS, if combined with the metabolic syndrome (MetS), might have a synergistic effect on low-density lipoprotein (LDL) particle size.

**Methods:**

Carotid intima-media thickness (IMT) and plaque formation, and HS were diagnosed ultrasonographically, and the MetS was diagnosed using the ATP III criteria in 274 healthy workers (mean age ± SD, 43.5 ± 7.1 yrs). LDL particle size was measured with density gradient ultracentrifugation, and subfractions were classified as large, buoyant LDL I (27.2~28.5 nm) and small, dense LDL III (24.2~25.5). All participants were grouped into three categories: control, subjects with HS alone and those with both HS and the MetS.

**Results:**

The subjects with HS alone were 84 (30.7%), whereas those with HS and the MetS were 46 (16.8%). LDL peak particle sizes showed significant negative correlations with carotid mean IMTs. LDL peak particle size and LDL I (%) decreased significantly in the HS, showing the lowest values in the subjects with both HS and the MetS, and their association was independent, even adjusted for potential confounders. LDL III also showed independent associations across the groups.

**Conclusion:**

HS alone was more prevalent than HS combined with the MetS in general population. For the patients with HS alone, LDL particle size and carotid atherosclerosis were found to fall in the middle of the control and those with both HS and the MetS.

## Introduction

Atherosclerosis and its relevant vascular events including cardiovascular disease (CVD), stroke, and peripheral arterial disease (PAD) have become a leading cause of disability and mortality in modern society [1]. Increasing trends of the vascular diseases are universal both in developed and developing countries [2]. A lifestyle summarized as a lack of physical activity and moderate-to-high intake of calories seems to be one of the most important causes of rapidly increasing prevalence of the metabolic syndrome (MetS) [3], type 2 diabetes mellitus (DM) [4], dyslipidemia [5], and eventually atherothrombotic diseases [2].

The sedentary lifestyle made people more dyslipidemic; atherogenic dyslipidemia, consisting of high triglyceride (TG), low high-density lipoprotein (HDL) cholesterol, and small low-density lipoprotein (LDL) particle size [6]. Smaller LDL particle size than normal has been known to be closely associated with the MetS [7], insulin resistance [8], CVD [9], and PAD [10]. LDL particle size was reported to be influenced by lipid-lowering agents [11] as well as by physical exercise [12]. The well-known determinants of LDL particle size include TG and HDL cholesterol, both of which are the major components of the MetS [13].

In the present study, it is hypothesized that hepatic steatosis (HS) could be associated with variable degrees of atherogenic dyslipidemia, and both LDL particle size and carotid atherosclerosis might be exacerbated if HS was combined with the MetS. HS, the most frequent liver disorder, was reported to be an independent predictor of CVD [14]. According to the results obtained with a ultrasonographical diagnosis for HS and a definition of the MetS, all subjects in the study were grouped into control, subjects with HS alone, and those with both HS and the MetS, and examined for the hypothesis using a cohort sample of randomly selected hospital workers.

## Methods

### Study population

The present cross-sectional study was intentionally designed and executed to investigate relations between HS and the MetS. The detailed explanation about the design was reported previously [15]. In brief, according to the age- and sex-stratified random sampling, 334 hospital workers were randomly selected and among them, 135 men and 158 women (response rate of 87.7%) participated in the survey. All participants gave their written informed consents to the participation in this study, and the study was approved by the institutional research ethics committee.

### Data collection and measurements

The administered questionnaire was designed to determine the prior history of CVD, type 2 DM, hypertension, and medication usage. Information regarding alcohol-drinking status, as estimated by the frequency, duration, amount and kind of liquor consumed, was obtained, and the mean ethanol intake per day was calculated. Smoking status was classified into three categories: current smokers, ex-smokers, and non-smokers. Waist circumference was measured, with the subject standing and wearing no underwear, at the level midway between the lower rib margin and the iliac crest. Body mass index (BMI) was calculated by a computer as weight divided by height squared (kg/m^2^).

### Abdominal ultrasonography and definition of hepatic steatosis (HS)

All abdominal ultrasonographic scans were performed by one radiologist (Y. K. K) who was blinded to the patients' histories and laboratory results. His annual total number of cases of abdominal sonography has exceeded 5,000 during the past 8 years. Fatty liver measurements were made using a 3.5-MHz convex probe (Sequoia, Siemens Medical Solutions, Mountain View, CA) in all subjects. Hepatic steatosis was diagnosed by characteristic echo-patterns, according to the conventional criteria (i.e., evidence of a diffuse increase in echogenicity of the liver as compared with that of the kidney) [16]. The examiner also assessed the evidence of chronic hepatitis or liver cirrhosis, including hepatic nodularity, coarseness of liver parenchyma, and splenomegaly. Repeated measurements on the same subjects gave coefficients of variation (CV) of < 1% for the presence of fatty liver, as reported early [15].

### Definition of the metabolic syndrome (MetS)

The MetS was identified by the presence of three or more of the following five components, according to the modified criteria of the Third Adults Treatment Panel (modified ATP-III) of the National Cholesterol Education Program (NCEP), with waist cutoffs appropriate for Asian population [13]: 1) abdominal obesity (waist circumference ≥ 90 cm for men and ≥ 80 cm for women); 2) high blood pressure (≥ 130/85 mmHg or use of antihypertensives); 3) high TG (≥ 1.7 mmol/L or 150 mg/dL); 4) low HDL cholesterol (< 1.03 mmol/L or 40 mg/dL for men and < 1.3 mmol/L or 50 mg/dL for women); and 5) high fasting glucose (≥ 5.6 mmol/L or 100 mg/dL).

### Carotid intima-media thickness (IMT) and plaque formation

The measurement of the carotid IMT was conducted using a higher-frequency 7.0-MHz linear transducer (Sequoia; Siemens Medical Solutions, Mountain View, CA) with compound and harmonic imaging to reduce near-field artifacts [17]. The carotid IMT, a double-line pattern visualized by echotomography on the far wall of both distal common carotid arteries (CCA) and proximal internal carotid arteries (ICA), was measured in a region free of plaque, and their mean values were calculated. Atherosclerotic plaque was defined as a focal structure encroaching the arterial lumen of at least 0.5 mm or 50% of the surrounding IMT value, or demonstrated a thickness > 1.5 mm as measured from the media-adventitia interface to the intima-lumen interface [18]. Repeated measurements on the same objects (30 subjects) gave a CV < 6.2%.

### Biochemical investigations

Blood samples were collected in the morning before breakfast after an overnight fast. Serum biochemistries were assessed with a Hitachi 7600-110 analyzer (Hitachi High-Technologies Corporation, Tokyo, Japan). Most laboratory investigations were described previously in detail [15]. In addition, serum insulin was determined by the electrochemiluminescence immunoassay using Modular Analytics E170 (Roche Diagnostics GmbH, Mannheim, Germany), and insulin resistance was calculated by homeostasis model assessment for insulin resistance (HOMA-IR) score [19]. Serum TG and glucose were determined enzymatically (Roche Diagnostics GmbH, Mannheim, Germany). HDL cholesterol was measured enzymatically as cholesterol after selective disruption of HDL only (Daiichi Pure Chemicals Co., Ltd., Japan). Apolipoprotein A-I (apo A-I) and B (apo B) assays were analyzed by a Roche/Hitachi Modular P Chemistry analyzer that used an immunoturbidimetric assay (Roche Diagnostics GmbH, Mannheim, Germany). Plasma concentration of total homocysteine was measured by fluorescence polarization immunoassay (AxSYM, Abbott Laboratories, Abbott Park, IL).

### Measurement of LDL particle size

The peak particle size of LDL was measured by both a density gradient ultracentrifugation and a pore gradient lipoprotein system (CBS Scientific, Del Mar, CA) with commercially available non-denaturing 2-16% polyacrylamide gradient gels (Alamo Gels Inc., San Antonio, TX). Standard markers of polystyrene latex beads (36 nm), thyroglobulin (17 nm), apoferritin (12.2 nm) and catalase (10.4 nm) were used to estimate the relative migration rates of each band. The gels were scanned with a GS-800 Calibrated Imaging Densitometer (Bio-Rad Laboratories, Graz, Austria). LDL particle size was calculated with reference to the relative migration value of the standard markers [20]. For a quantitative subfraction analysis, LDL I (if LDL peak particle size was 27.2~28.5 nm) and LDL III (24.2~25.5 nm) were classified, and their proportions were used in the present study.

### Statistical Analysis

The descriptive data for the major characteristics of the aforementioned three categories were expressed as the mean ± standard deviation (SD) or percentage as appropriately. An analysis of variance (ANOVA) was used to determine the statistical differences in the continuous variables and the chi-square test for trend for categorical variables. For the LDL particle sizes, mean values and 95% confidence intervals (CI) according to the three categories and scatter plots with carotid IMTs were depicted. A general linear model was used to evaluate how adjusted mean values of LDL particle sizes and carotid atherosclerosis varied in the three categories. Bonferroni tests were applied to correct for multiple comparisons. Interaction terms like three categories x variables (i.e., sex) were created, and their significances were assessed. All statistical analyses were conducted using SPSS software version 16.0 (SPSS, Chicago, TX)

## Results

Among the 293 participants, 15 (5.1%) subjects who had the MetS with no combined HS and 4 (1.4%) who did not undergo abdominal ultrasonographic examinations were excluded from the present study. Finally, 125 men and 149 women (93.5%) were analyzed. Excluded subjects showed statistically significantly higher age than included ones (47.4 ± 7.6 vs. 43.5 ± 7.1 y, *p *= 0.022), but percentages of women did not show much difference (54.4% vs. 47.4%, *P *= 0.553).

According to the definitions for HS and the MetS, 84 subjects (30.7%) were categorized as the subjects with HS alone, 46 (16.8%) as those with both HS and the MetS, and the rest (144 subjects) as control, as shown in Table 1. Age was not significantly different in the three groups. A proportion of women was lowest in the subjects with both HS and the MetS, and the proportions of smokers and alcohol drinkers (more than 20 g/d) were significantly higher in the subjects with HS.

**Table 1:** Baseline characteristics of the subjects according to hepatic steatosis (HS) and the metabolic syndrome (MetS)

	Control	HS alone	HS with MetS	
Characteristics	(n = 144)	(n = 84)	(n = 46)	*P*^a^
Age, y	42.9 ± 7.1	43.7 ± 6.7	45.1 ± 7.9	0.178
Women, %	68.1	47.6	23.9	< 0.001
Smoking, ex- and current, %	26.1	39.3	56.5	< 0.001
Alcohol consumption (≥20 g/d), %	15.3	23.8	45.7	< 0.001
ALT, IU/L	18.4 ± 8.8	24.5 ± 14.1**	37.5 ± 24.5***^†^	< 0.001
Ferritin, ng/ml	51.7 ± 47.5	74.7 ± 62.3*	131.6 ± 106.3***^†^	< 0.001
Body mass index, kg/m^2^	21.8 ± 2.2	24.3 ± 2.3***	26.0 ± 2.1***^†^	< 0.001

Values are mean ± SD unless noted otherwise. Components of the metabolic syndrome are not expressed. ALT; alanine aminotransferase.

^a ^*P *values by analysis of variance (ANOVA) or chi-square test for trend.

*P < 0.05, ** *P *< 0.01, *** *P *< 0.001; compared with the control (Bonferroni comparison).

^† ^*P *< 0.001; compared with the HS alone (Bonferroni comparison).

Total cholesterol, LDL cholesterol, and apo B were significantly elevated in the subjects with HS alone, even adjusted for age, sex, alcohol drinking and smoking, as shown in Table 2. HOMA-IR showed significantly elevated values across the groups with the highest value in the subjects with both HS and the MetS, and fasting insulin levels showed a similar trend as HOMA-IR and reached a borderline significance.

**Table 2:** Adjusted mean values (± SE) of variables on lipid and insulin resistance

Lipid profiles		Control	HS alone	HS with MetS^a^	*P *value (Between groups)	*P *value (Linear trend)
Total cholesterol, mmol/L		4.54 ± 0.06	4.92 ± 0.08**	4.88 ± 0.12	0.012	0.002
LDL cholesterol, mmol/L		2.74 ± 0.06	3.09 ± 0.07**	3.00 ± 0.10*	0.001	0.019
Triglyceride, mmol/L		1.11 ± 0.05	1.48 ± 0.07***^††^	2.20 ± 1.00***^††^	< 0.001	< 0.001
HDL cholesterol, mmol/L		1.34 ± 0.02	1.23 ± 0.03**	1.09 ± 0.04***^†^	< 0.001	< 0.001
Lipoprotein(a), mmol/L		0.71 ± 0.05	0.77 ± 0.09	0.67 ± 0.09	0.632	0.699
Apo B, g/L		0.77 ± 0.02	0.88 ± 0.02**	0.91 ± 0.03**	< 0.001	< 0.001
Apo A-I, g/L		1.27 ± 0.03	1.23 ± 0.03	1.24 ± 0.05	0.623	0.537
**Plsama total homocysteine (tHcy) and HOMA-IR**						
Plasma tHcy, μmol/L		6.9 ± 0.2	7.2 ± 0.3	7.2 ± 0.4	0.593	0.471
Fasting insulin, μU/ml		5.3 ± 0.5	6.8 ± 0.6	7.2 ± 0.9	0.083	0.056
HOMA-IR		1.3 ± 0.1	1.7 ± 0.2	1.9 ± 0.2*	0.030	0.014

Adjusted for age, sex, alcohol drinking (≥20 g/d), and smoking. LDL, low-density lipoprotein; HDL, high-density lipoprotein; apo B, apolipoprotein B; apo A-I, apolipoprotein A-I; HOMA-IR; homeostasis model assessment for insulin resistance.

*P < 0.05, ** *P *< 0.01, *** *P *< 0.001; compared with the control (Bonferroni adjustment for multiple comparisons).

^†^*P *< 0.05, ^†† ^*P *< 0.001; compared with the HS alone (Bonferroni adjustment for multiple comparisons).

**Figure 1:**
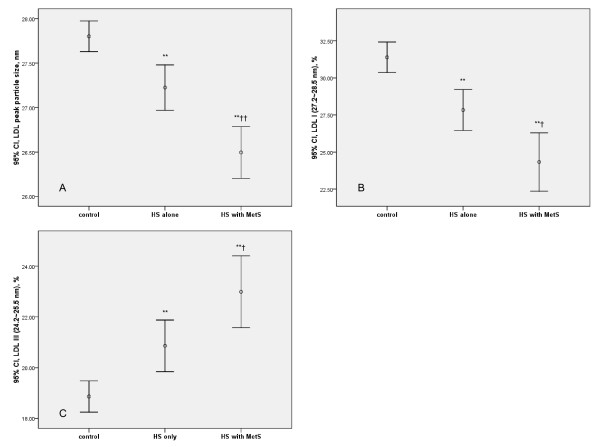
**LDL peak particle size and subfractions in hepatic steatosis (HS) according to its combination with/without the metabolic syndrome (MetS)**. A. Mean values (with 95% CIs) of LDL peak particle size (nm), B. Large, buoyant LDL subfraction I (%), and C. Small, dense LDL subfraction III (%): All three values showed significant linear associations with the three categories (*P *< 0.001). * *P *< 0.01, **P < 0.001; compared with the control (Bonferroni comparison). ^† ^*P *< 0.05, ^†† ^*P *< 0.01; compared with the HS alone.

**Figure 2:**
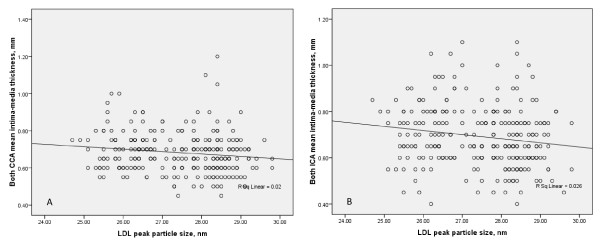
**Scatter plots of carotid intima-media thickness (IMT) and LDL peak particle size**. A. A scatter plot of both CCA mean IMT and LDL peak particle size, *r *= -0.14, *P *= 0.021; B. A scatter plot of both ICA mean IMT and LDL peak particle size, *r *= -0.16, *P *= 0.010.

As for LDL peak particle size and the proportions of large, buoyant LDL particles (LDL I, 27.2~28.5 nm), the control group had the largest values, whereas the HS and MetS combined group had the lowest values, as shown in Figures 1A and 1B. Regarding the proportions of small, dense LDL particles (LDL III, 24.2~25.5 nm), the control group had the least values, whereas the combined group had the largest values as shown in Figure 1C. Scatter plots of the LDL peak particle size and carotid IMTs of both mean CCA and ICA showed significant negative correlations, as depicted in Figures 2A and 1B. The correlation coefficients (*r*) were -0.14 and -0.16 for mean CCA and ICA, respectively. As for the effect modifiers for the association presented in Figures 1 and 2, neither sex nor other factors like alcohol consumption and nonalcoholic fatty liver disease (NAFLD) showed significant interaction terms (all *P *for interaction > 0.1).

Both mean CCA and ICA IMTs showed significantly increased values across the three groups, with the highest IMTs in the combined group adjusting for age, sex, alcohol drinking and smoking, as shown in Table 3. The percentage of carotid plaque showed similar patterns with carotid IMTs, with the highest percentage in the combined group.

**Table 3:** Adjusted mean values (± SE) of carotid intima-media thickness (IMT) and plaque

	Control	HS alone	HS with MetS	*P *value (Between groups)	*P *value (Linear trend)
Carotid mean IMT					
Both CCA mean IMT, mm	0.67 ± 0.01	0.68 ± 0.01	0.72 ± 0.02*	0.042	0.013
Both ICA mean IMT, mm	0.68 ± 0.01	0.69 ± 0.01	0.74 ± 0.02*	0.032	0.015
Carotid plaque					
Carotid plaque, crude, %	11.1 ± 0.3	14.3 ± 0.4	32.6 ± 0.5**^†^	0.002	0.001
Carotid plaque, adjusted, %	12.5 ± 0.3	13.8 ± 0.4	29.8 ± 0.5*^†^	0.017	0.007

Adjusted for age, sex, alcohol drinking (≥20 g/d), and smoking. CCA; common carotid artery, ICA; internal carotid artery.

*P < 0.05, ** *P *< 0.01; compared with the control (Bonferroni adjustment for multiple comparisons).

^† ^*P *< 0.05; compared with the HS alone (Bonferroni adjustment for multiple comparisons).

The associations between the LDL peak particle size and the three groups were independent, even adjusted for the potential confounders including BMI, HOMA-IR, and apo B, as shown in Table 4. The subjects with HS alone showed independently lower values of LDL peak particle size and large, buoyant LDL I (%) than the control. As for small, dense LDL III (%), a difference between the subjects with HS alone and the control lost significance after an adjustment. The subjects with both HS and MetS showed the lowest values for LDL peak particle size and LDL I (%), and the highest values for LDL III (%) among the three categories.

**Table 4:** Adjusted mean values (± SE) of LDL particle sizes

	Control	HS alone	HS with MetS	*P *value (Between groups)	*P *value (Linear trend)
LDL peak particle size, nm					
Model 1	27.6 ± 0.1	27.2 ± 0.1*	26.8 ± 0.2**	0.001	< 0.001
Model 2	27.6 ± 0.1	27.3 ± 0.1	26.9 ± 0.2**	0.009	0.003
LDL I (27.2~28.5 nm), %					
Model 1	30.8 ± 0.6	27.9 ± 0.7*	25.9 ± 1.1**	0.001	0.001
Model 2	30.5 ± 0.6	28.0 ± 0.7*	26.7 ± 1.0*	0.005	0.004
LDL III, (24.2~25.5 nm), %					
Model 1	19.3 ± 0.4	20.8 ± 0.5	22.2 ± 0.8**	0.006	0.002
Model 2	19.5 ± 0.4	20.8 ± 0.5	21.7 ± 0.7*	0.026	0.013

Model 1 included age, sex, alcohol drinking (≥20g/d), smoking, BMI, and HOMA-IR.

Model 2 included variables in the model 1 plus total cholesterol, LDL cholesterol, and apo B.

* P < 0.05, ** P < 0.01; compared with the control (Bonferroni adjustment for multiple comparisons).

## Discussion

The present study showed independent associations of both hepatic steatosis (HS) and the metabolic syndrome (MetS) with LDL particle size, even adjusted for the potential confounders including apo B and HOMA-IR. And the significant linear trends of LDL particle sizes supported the original hypothesis that atherogenic dyslipidemia could be exacerbated if MetS combined with HS. The present study further showed that HS itself, even when HS was not combined with the MetS, showed significant and independent differences as for LDL particle sizes compared with the control. Until now, LDL particle size in HS has been reported only in patients with type 2 DM [21], but not yet in general population.

LDL particle size, in nature, is closely related with serum levels of TG, HDL cholesterol, and insulin resistance [7]. As large amounts of plasma TG are carried or transported by very low density lipoprotein (VLDL) from liver, TG-rich HDL cholesterol which is made by an action of cholesterol ester transfer protein (CETP) [22] undergoes hydrolysis by lipoprotein lipase (LPL) and eventually is degraded by kidney. Then, TG-rich lipoproteins (TRLs) depletes TG by lipolysis, thus giving rise to small, dense LDL particles [23]. As for cardiovascular risk, both quality (i.e., the question of how small LDL peak particle size is) and quantity (i.e., the percentage of small LDL III) of LDL particle size were reported to be important equally and additively [24], as shown in Table 3.

The smaller LDL particle size in the subjects with HS alone than the control could be explained with a concept of hepatic insulin resistance, which occurred primarily [25] and more evidently [26] than peripheral insulin resistance. Hepatic fat accumulation in HepG2 cells was found to induce serine phosphorylation of insulin receptor substrate (IRS)-1 and endoplasmic reticulum (ER) stress, as previously reported by the authors [27]. The ER stress, in turn, led to the suppression of insulin receptor signaling, causing hepatic insulin resistance and an enhanced TG synthesis [28]. The HS alone in the present study could be regarded as hepatic insulin resistance with no evident peripheral insulin resistance. Contrary to LDL particle sizes, both fasting insulin and HOMA-IR, markers of peripheral insulin resistance, did not show any significant differences between the control and the subjects with HS alone, as shown in Table 2.

NAFLD, which can be defined by alcohol consumption less than 20 g/d (excluding hepatitis B virus and hepatitis C virus infections) [29], has been reported to be closely related with subsequent metabolic diseases [30]. In the present population study, however, the association between HS and LDL particle size was not modified by alcohol consumption or NAFLD. A recent study about fatty liver index reported significant associations among high values of the index, atherosclerosis, and insulin resistance, where fatty liver was not divided into alcoholic or nonalcoholic subtypes either [31].

The present study has several limitations. First, HS was not examined histologically, but diagnosed ultrasonographically. Although the combination of HS and the MetS might result in advanced pathologic findings of liver like steatohepatitis, the authors were unable to define such changes exactly. However, the abdominal ultrasonographic examination was sufficient to detect the presence of fatty liver for research purposes [16]. The diagnosis of fatty liver using ultrasonography was reported to have a somewhat low sensitivity and high specificity [32], thus the present control group was believed to be selected appropriately. Second, the present study did not collect the information on food or nutrient consumption such as niacin [33]. So the present study could not delineate whether the LDL particle size was modified by the additional nutrient supplement. Third, the present result did not adjust further with TG or HDL-cholesterol, although they were known as the most important determinants of LDL particle sizes. But, the three categorizations in the study used the MetS which included TG and HDL cholesterol as important components for a definition [13]. Hence, the additional adjustment of the two components seemed to be an overcorrection in the present model. Last, the present results were obtained from a cross-sectional design. Accordingly a subsequent longitudinal study may be warranted to establish a biologic plausibility. Currently, a new study is underway to perform a chylomicron-clearance test for the same subjects involved in the present study.

In summary, the present study demonstrated that LDL peak particle size was negatively correlated with carotid IMT and plaque, and that hepatic fatty infiltration (HS) was independently associated with the LDL particle size in terms of both quantity and quality, irrespective of the co-existence of the MetS. HS, even when there is a subtle change to be recognized, should be regarded and monitored carefully as an early hepatic manifestation of atherogenic dyslipidemia.

## Conflicts of interests

The authors declare that they have no competing interests.

## Authors' contributions

DSK and YKK participated in data collection and reviewed the manuscript. DSK, HJC, TSP, and YIC conceived the study and reviewed the manuscript. SKJ participated in data collection, drafted, and reviewed the manuscript. All authors read and approved the final manuscript.
